# Enhancing Charge Trapping Performance of Hafnia Thin Films Using Sequential Plasma Atomic Layer Deposition

**DOI:** 10.3390/nano14201686

**Published:** 2024-10-21

**Authors:** So-Won Kim, Jae-Hoon Yoo, Won-Ji Park, Chan-Hee Lee, Joung-Ho Lee, Jong-Hwan Kim, Sae-Hoon Uhm, Hee-Chul Lee

**Affiliations:** 1 Department of Advanced Materials Engineering, Tech University of Korea, Siheung 15073, Republic of Korea; swkim9193@tukorea.ac.kr (S.-W.K.); pullat@tukorea.ac.kr (J.-H.Y.); wonji1221@tukorea.ac.kr (W.-J.P.); leech5026a6@tukorea.ac.kr (C.-H.L.); rmfladl91@tukorea.ac.kr (J.-H.K.); 2 Korea Evaluation Institute of Industrial Technology, Seoul 06152, Republic of Korea; plasma@keit.re.kr; 3 EN2CORE Technology Inc., Daejeon 18469, Republic of Korea; shuhm@en2core.com

**Keywords:** charge-trapping memory, sequential plasma ALD, interface trapping density, memory window, HfO_2_, retention

## Abstract

We aimed to fabricate reliable memory devices using HfO_2_, which is gaining attention as a charge-trapping layer material for next-generation NAND flash memory. To this end, a new atomic layer deposition process using sequential remote plasma (RP) and direct plasma (DP) was designed to create charge-trapping memory devices. Subsequently, the operational characteristics of the devices were analyzed based on the thickness ratio of thin films deposited using the sequential RP and DP processes. As the thickness of the initially RP-deposited thin film increased, the memory window and retention also increased, while the interface defect density and leakage current decreased. When the thickness of the RP-deposited thin film was 7 nm, a maximum memory window of 10.1 V was achieved at an operating voltage of ±10 V, and the interface trap density (D_it_) reached a minimum value of 1.0 × 10^12^ eV^−1^cm^−2^. Once the RP-deposited thin film reaches a certain thickness, the ion bombardment effect from DP on the substrate is expected to decrease, improving the Si/SiO_2_/HfO_2_ interface and thereby enhancing device endurance and reliability. This study confirmed that the proposed sequential RP and DP deposition processes could resolve issues related to unstable interface layers, improve device performance, and enhance process throughput.

## 1. Introduction

With recent advancements in ultra-miniaturization and high integration of semiconductor devices, thin-film technology has become increasingly prominent in enhancing the performance of next-generation memory devices. In particular, memory devices utilizing HfO_2_-based materials are gaining significant attention for their performance and stability [1,2,3]. Compared to other non-volatile memories such as magnetic random-access memory (MRAM), phase-change random-access memory (PCRAM), and ferroelectric random-access memory (FRAM), NAND flash memory has relatively slower speeds and issues with durability. However, it offers significant advantages in terms of memory cost and capacity, making it widely applicable [4,5]. Reducing the thickness of the charge-trapping layer (CTL) helps to further increase the integration density of NAND flash memory devices. Therefore, it is essential to develop materials that can replace the traditional silicon nitride-based CTL [6,7,8]. HfO_2_ is being highlighted as a potential material for CTL in next-generation NAND flash memory owing to its high trap density, large band offset with silicon, and small equivalent oxide thickness (EOT) [9,10,11]. Currently, atomic layer deposition (ALD) is the most widely used process for forming such ultra-thin films in the order of a few nanometers [12,13,14,15].

In previous studies, our research team analyzed the operational characteristics of charge-trapping memory (CTM) devices fabricated by depositing HfO_2_ CTL thin films using remote plasma ALD (RPALD) and direct plasma ALD (DPALD). The results indicated that CTM devices utilizing remote plasma (RP)-HfO_2_ thin films exhibited superior charge trapping efficiency and device reliability [16]. This was because, although direct plasma (DP)-HfO_2_ thin films contained more internal oxygen vacancies than RP-HfO_2_ thin films, the deposition of HfO_2_ thin films using DP resulted in ion bombardment damage to the Si substrate, leading to the formation of unstable interface layers and degradation of memory characteristics [17,18,19]. Conversely, the RP deposition method has the advantage of minimizing substrate damage and interface reactions caused by plasma, as it does not directly apply plasma to the substrate. However, RPALD has the drawback of having significantly longer reactant injection and plasma discharge times than DPALD, resulting in reduced throughput [20,21,22]. Therefore, we propose a new process technology to prevent the degradation of thin-film characteristics due to substrate damage and unstable interface layers, while improving process throughput for the fabrication of highly reliable memory devices.

In this study, we closely analyzed the phenomenon of memory-characteristic degradation due to DP-induced substrate damage and interface reactions. To mitigate the degradation, we deposited HfO_2_ thin films through a new ALD process that utilized alternating RP and DP. Initially, a thin film of a certain thickness was formed using RP, followed by further deposition using DP until the target thickness was achieved. Using the deposited HfO_2_ thin films, we fabricated CTM devices with a MAHOS (Au/Al_2_O_3_/HfO_2_/SiO_2_/p-Si) structure. By electrically characterizing the CTM devices, we investigated the effect of Si/SiO_2_/HfO_2_ interface characteristics on the memory properties of the devices based on the thickness ratio of the RP/DP sequentially deposited thin films.

## 2. Materials and Methods

**Figure 1:**
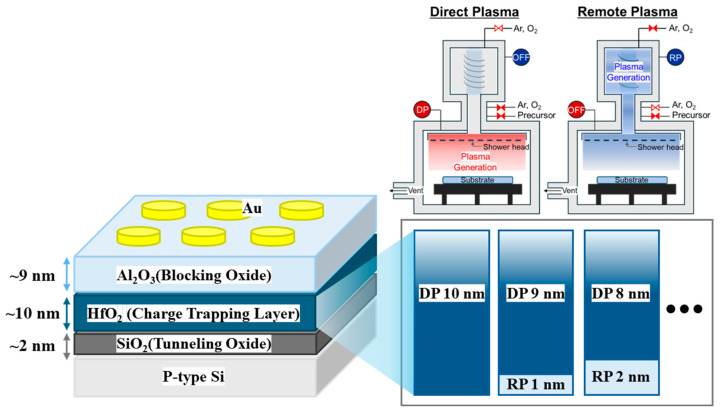
Schematic of the CTM device and equipment deposited using sequential remote plasma (RP) and direct plasma (DP).

In this study, p-type (100) silicon wafers were used as substrates. The substrates were cleaned using RCA cleaning and then immersed in buffered oxide etchant for approximately 30 s to remove the native oxide layer on the surface. For thin-film deposition on the substrate, we used a plasma-enhanced ALD (PEALD, iOV-dx2, iSAC Research, Daejeon, Korea) system. The RP was generated using a separate remote plasma system (RPS, En2ra-RPS, EN2CORE Technology, Daejeon, Korea), which was located in a space separate from the main chamber, whereas the DP was generated by the plasma generator within the PEALD system. For the deposition of HfO_2_ thin films, tetrakis (ethylmethylamino) hafnium (TEMAH, iChems, Hwaseong, Korea) was used as the Hf precursor. As shown in Figure 1, each cycle was repeated such that the thickness ratio of the thin films deposited using RP and DP was x:10 − x (x = 0–10 nm), forming a single HfO_2_ thin film with a total thickness of 10 nm. At this time, the interface layer formed by the interface reaction between the Si and HfO_2_ thin films was utilized as a tunneling oxide (TO) layer. During deposition, the process temperature for both RP and DP processes was maintained at 200 °C, and O_2_ was used as the reactive gas. On top of the 10 nm HfO_2_ thin film, an Al_2_O_3_ thin film was formed through DPALD as the blocking oxide (BO) layer to fabricate the CTM device. The top electrode was fabricated using the lift-off method to create a 50 nm thick Au electrode, and Au was deposited at room temperature for 3 min via DC magnetron sputtering. Finally, post-deposition annealing was conducted at 400 °C for 20 min in an N_2_ atmosphere using a rapid thermal annealing system.

X-ray diffraction (XRD, SmartLab, Rigaku, Tokyo, Japan) was utilized to analyze the crystallinity of the HfO_2_ thin films deposited using RP/DP sequential plasma ALD. The chemical bonding states of the Si/SiO_2_/HfO_2_ interface layer were examined using X-ray photoelectron spectroscopy (XPS, AXIS-NOVA, Manchester, UK). Additionally, to analyze the electrical and reliability characteristics of the fabricated MAHOS-structured CTM, measurements and analyses were conducted using a semiconductor parameter analyzer (4200-SCS, Keithley, Cleveland, OH, USA) connected to a microprobe station (APX-6B, WIT Co., Suwon, Korea).

## 3. Results and Discussion

**Figure 2:**
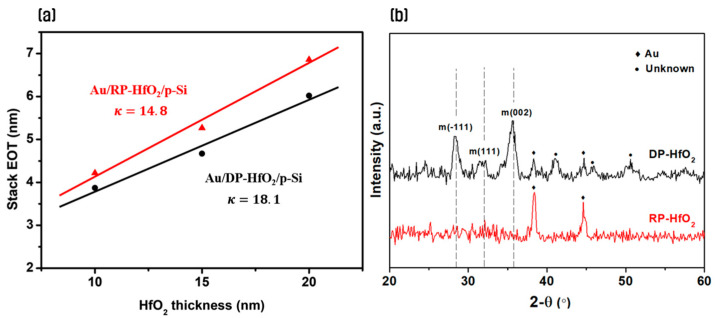
(**a**) Variation of EOT with HfO_2_ deposition thickness and (**b**) X-ray diffraction pattern analysis results of DP- and RP-HfO_2_ thin films.

Figure 2 shows the results of XRD pattern analysis of HfO_2_ thin films according to the equivalent oxide thickness (EOT) and deposition method based on HfO_2_ deposition thickness in a metal-oxide-semiconductor structure. The dielectric constant of the HfO_2_ thin films was calculated using Equation (1) based on the measured capacitance values from the C-V accumulation region of the MAHOS samples, in which HfO_2_ thin films were deposited using RP and DP, respectively (Figure 2a). Based on these values, the EOT of the HfO_2_ thin films was calculated using Equation (2).

(1) $$\kappa_{{HfO}_{2}}=\frac{C_{{HfO}_{2}}\times t_{{HfO}_{2}}}{\varepsilon_{0}}$$

(2) $$EOT=t_{{HfO}_{2}}\frac{\kappa_{{SiO}_{2}}}{\kappa_{{HfO}_{2}}}$$

Here, κ_HfO2_ represents the dielectric constant of HfO_2_, C_HfO2_ is the measured capacitance per unit area, t_HfO2_ is the actual thickness of the HfO_2_ thin films, ε_0_ is the permittivity of vacuum, and κ_SiO2_ is the dielectric constant of SiO_2_ [23,24]. The results indicated that the dielectric constant was lower when using RP (κ = 14.8) compared to DP (κ = 18.1). As can be observed from the XRD results in Figure 2b, the HfO_2_ thin film deposited using RP shows an amorphous state with minimal HfO_2_ crystallization, whereas the thin film deposited using DP exhibits monoclinic crystal phases around 28°, 32°, and 36° [25,26]. This suggests that the thin film deposited using DP has a higher dielectric constant due to crystallization than that deposited using RP. Although the HfO_2_ thin film deposited using RP shows a larger EOT, it is expected to be advantageous for reducing leakage current and suppressing charge de-trapping phenomena owing to its amorphous state, unlike the thin film deposited using DP [27,28,29,30]. Therefore, despite a slight EOT loss, we aimed to investigate the feasibility of leveraging the high-quality interface characteristics and improved electrical properties of high-k HfO_2_ thin films for a CTL by controlling the thickness ratio of HfO_2_ thin films deposited using the sequential RP and DP deposition processes.

**Figure 3:**
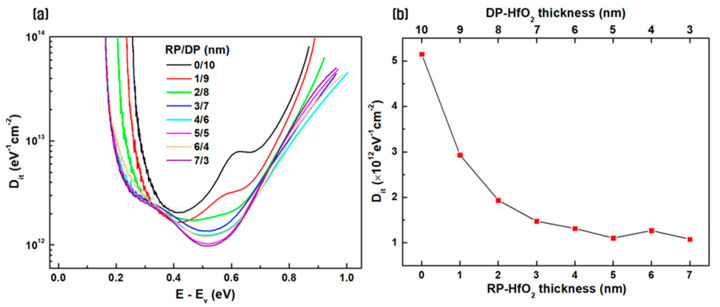
(**a**) Distribution of interface trap density (D_it_) within the Si bandgap and (**b**) calculated D_it_ values at midgap for different thickness ratios of thin films deposited via the sequential remote plasma (RP) and direct plasma (DP) processes.

Figure 3 shows the interface trap density for different thickness ratios of thin films deposited using the sequential plasma ALD process (RP/DP). The trap density was calculated using the high-low-frequency capacitance method proposed by Castagne and Vapaille [31]. Figure 3a shows the distribution of interface trap density in the energy region of Si. The surface potential with respect to the gate voltage was obtained using the Berglund integral, and the D_it_ distribution within the Si bandgap was calculated accordingly [32,33]. It was confirmed that as the thickness of the thin film initially deposited with RP increased, the interface trap density per unit area decreased across the energy region. This is attributed to the occurrence of deposition and plasma discharge in one space when deposited using only DP, damaging the substrate and thin film due to ion bombardment and creating an unstable interface layer and defects. The ion bombardment effect at the interface is mitigated when the thin film is initially deposited using RP, decreasing the interface trap density [22,34]. In Figure 3b, the D_it_ value at the midgap (0.56 eV) of the silicon bandgap (1.12 eV) is shown. The D_it_ value decreases to below 1.31 × 10^12^ eV^−1^cm^−2^ when the thickness of RP-HfO_2_ exceeds 4 nm, which is more than four times lower than the D_it_ value of a 10 nm HfO_2_ thin film deposited using only DP. In a previous study, the D_it_ value of the 10 nm HfO_2_ thin film deposited only using RP was confirmed to be 1.18 × 10^12^ eV^−1^cm^−2^ [16]. Therefore, a saturating trend is observed as the thickness of the RP-HfO_2_ film increases, and it is expected that the influence of DP on the interface significantly decreases beyond a film thickness of 4 nm.

**Figure 4:**
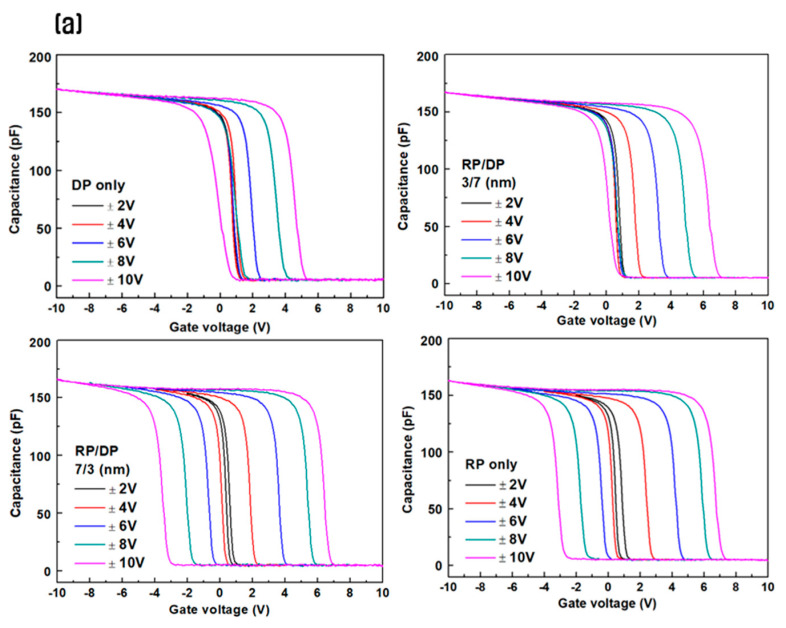

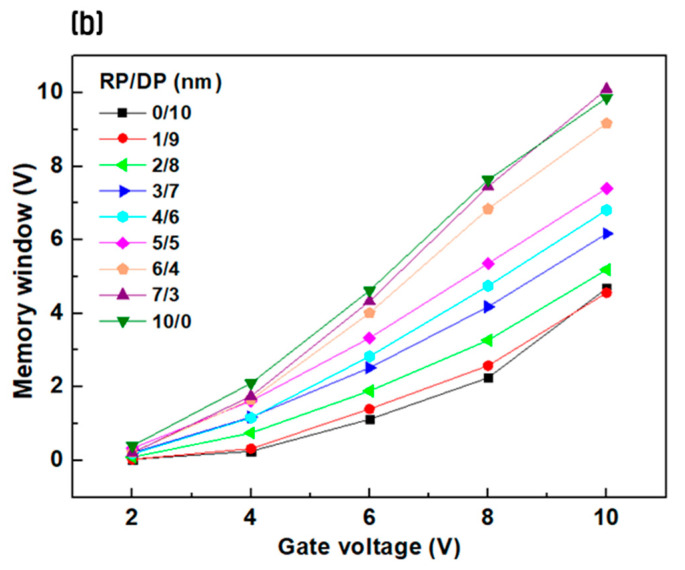
Comparison of (**a**) C-V curves and (**b**) memory window of HfO_2_ CTM deposited using the sequential remote plasma and direct plasma processes.

Figure 4 compares the C-V measurement results and the size of the memory window for HfO_2_ CTM according to the thickness ratio of the thin film layers deposited using the sequential RP and DP processes. The measurements were conducted at 1 MHz using a forward and reverse sweeping method. In the C-V curve shown in Figure 4a, a typical counterclockwise hysteresis caused by the trapping of mobile carriers, such as electrons and holes, can be observed [35]. The memory window value was calculated from the difference in V_FB_ (flat-band voltage) between the program and erase states in the C-V measurement results, and it is presented in Figure 4b. As the sweeping voltage increases, the thin film initially deposited using RP exhibits a significant increase in the memory window with increasing thickness compared to the thin film deposited solely using DP at a thickness of 10 nm. Additionally, for the device based on the 10 nm HfO_2_ thin film deposited only using DP, a memory window of 4.68 V is observed at an operating voltage of ±10 V. However, as the thickness of the thin film initially deposited using RP increases, the memory window gradually increases, reaching a maximum value of 10.1 V at an operating voltage of ±10 V when the RP-deposited thin film is 7 nm thick. As shown in Figure 3, the interface defect density is higher when only DP is used for deposition. The presence of interface defects likely hindered the trapping and de-trapping of charges through TO, and the concentration of traps in specific defect regions may have contributed to the reduced size of the memory window [36,37,38].

**Figure 5:**
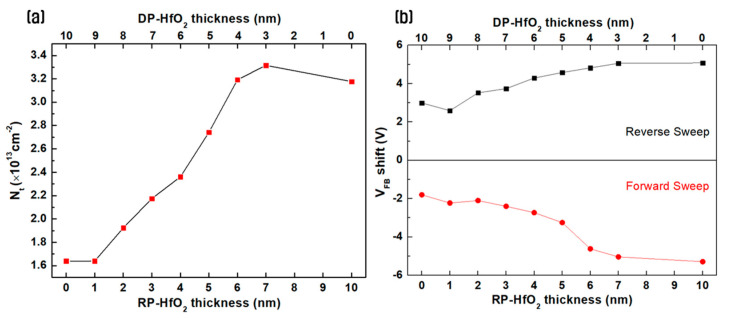
(**a**) Charge density in the thin film and (**b**) V_FB_ shift results of HfO_2_ CTM according to the thickness ratio of the thin film layers deposited using the sequential remote plasma and direct plasma processes.

Figure 5a shows the charge density in the thin film according to the thickness ratio of the thin films deposited through the sequential RP and DP processes. The charge trapping density per unit area (N_t_) was calculated using the equation N_t_ = (C_ox_ × ∆V_FB_)/qA at the point where the memory window saturated as the sweeping voltage increased. Here, C_ox_ is the capacitance in the accumulation region, ∆V_FB_ is the memory window, q is the electron charge, and A is the effective area of the top electrode [39]. N_t_ showed a value of 1.6 × 10^13^ cm^−2^ when the deposition was performed using only DP, and it continuously increased as the thickness of the RP-deposited film increased. When the thickness of the RP-deposited film was 7 nm, N_t_ reached 3.31 × 10^13^ cm^−2^, which is approximately twice the maximum value, indicating excellent charge trapping efficiency. Figure 5b shows the V_FB_ shift results according to the thickness ratio of the thin films deposited through the sequential RP and DP processes. As the thickness of the initially RP-deposited film increased, the V_FB_ shift increased during the reverse sweep, indicating an increase in the number of effective sites within the CTL where charges can be repeatedly trapped and de-trapped [38,40]. Additionally, during the forward sweep, hole trapping gradually increased as the thickness of the RP-deposited film increased up to a thickness of 5 nm and then rapidly increased after x = 6 nm. This result suggests that up to x = 5 nm, the ion bombardment effect from the subsequent DP deposition affected the underlying TO, reducing the hole-trapping efficiency. From x = 6 nm, the ion bombardment effect from DP disappeared, resulting in a V_FB_ shift similar to that of electron trapping. Therefore, it is expected that the Program/Erase (P/E) operation of the device can be smoothly performed when the thickness of the RP-deposited film is higher than or equal to 6 nm.

**Figure 6:**
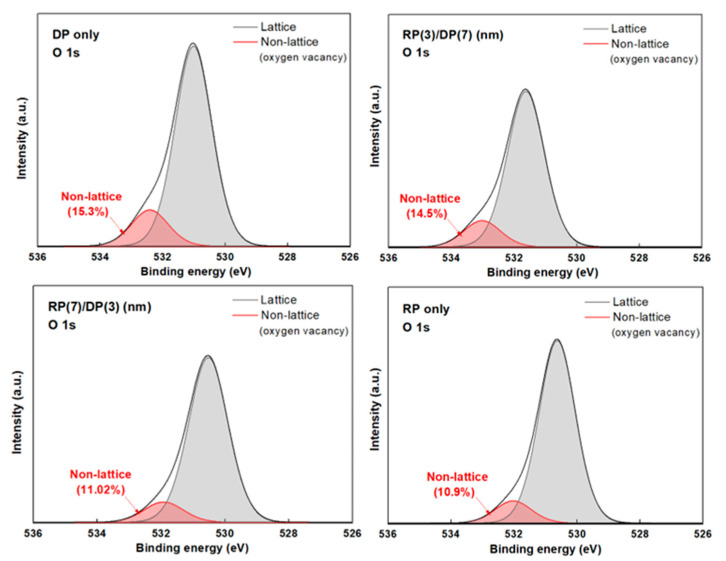
Comparison results of O 1s narrow scan XPS patterns according to the thickness ratio of thin film layers deposited using the sequential remote plasma and direct plasma processes.

Figure 6 shows the degree of defects at the interface according to the thickness ratio of the thin film layers deposited through the sequential RP and DP processes. To confirm the results, XPS analysis was performed near the Si/SiO_2_/HfO_2_ interface to measure the ratio of the number of lattice bonds to that of non-lattice bonds at the interface. The non-lattice ratio, which indicates oxygen vacancies as one of the defect factors, was 15.3% in the XPS O1s peak spectra when only DP was used. When the thickness ratio of the RP-deposited film was 3 nm and 7 nm, the non-lattice ratio gradually decreased to 14.5% and 11.02%, respectively. The non-lattice ratio was 10.9% when only RP was used for deposition. This result is consistent with previous research findings that reported that the initial deposition of the thin films using RP reduces ion impact damage, thereby decreasing oxygen vacancies near the interface and forming a stable interface [16].

**Figure 7:**
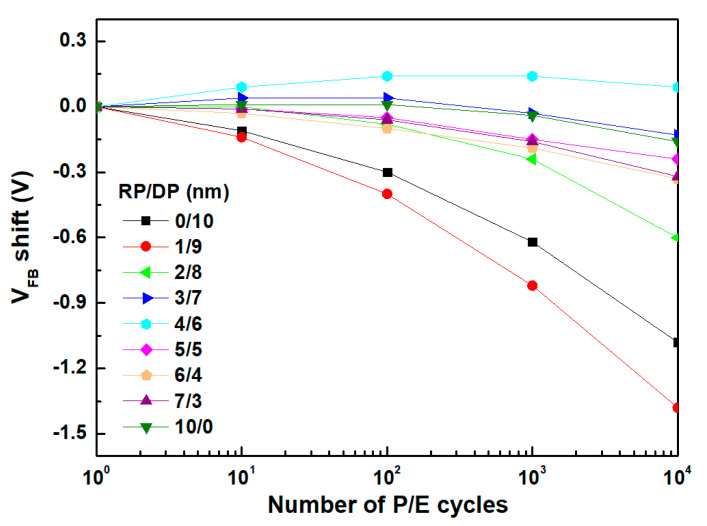
Endurance characteristics measurement results of HfO_2_ charge-trapping memory (CTM) deposited using the sequential remote plasma and direct plasma processes.

Figure 7 shows the evaluation results of V_FB_ shift characteristics according to P/E (Program/Erase) cycling to confirm the extent of device degradation based on the thickness ratio of thin films deposited using the sequential RP and DP processes. The P/E cycling was performed using a pulse train of amplitude and period ±8 V and 10 ms, respectively. For RP-deposited films with a thickness in the range of 0–2 nm, the overall V_FB_ shift was clearly observed to be in the negative direction. Generally, V_FB_ shift degradation is attributed to the failure of de-trapping electrons trapped due to the degradation of the Si/TO interface, TO, and CTL during P/E cycling, leading to a decrease in erase speed at the same gate voltage. This is likely because, during P/E cycling, thinner RP-deposited films result in unstable interface layers with many interface trap charges [31,32,33,34,35,36,37,38,39,40,41,42,43,44]. Therefore, it was confirmed that initially depositing a thin film of a certain thickness using RP, followed by DP deposition, enhances the reliability of the device during actual memory operation cycling.

**Figure 8:**
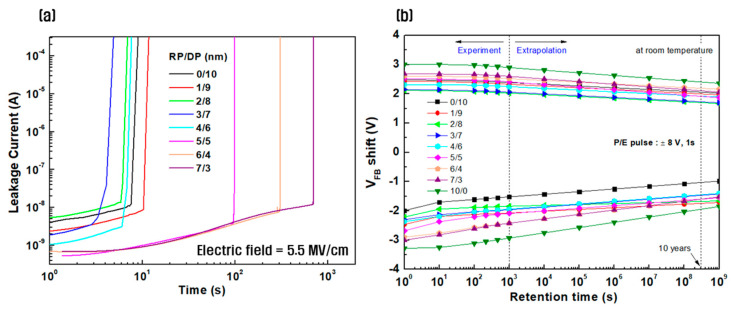
Evaluation results of (**a**) TDDB and (**b**) memory retention characteristics of HfO_2_ charge-trapping memory (CTM) deposited using the sequential remote plasma and direct plasma processes.

Figure 8a shows the results of a time-dependent dielectric breakdown (TDDB) test conducted on HfO_2_ CTM devices deposited using the sequential RP and DP processes to predict the reliability of the high-k HfO_2_ thin film. The measurements were conducted at room temperature, and a high voltage of 5.5 MV/cm was applied to the device to observe changes in leakage current characteristics over time. For thin films deposited with an initial RP thickness of x = 0–4 nm, weak endurance was observed within approximately 10 s. However, beyond x = 5 nm, the endurance time increased, and the initial leakage current gradually decreased as the thickness of the RP-deposited film increased. Generally, defects at the interface concentrate electrical stress, accelerating thin film degradation and affecting stable charge storage and reduction of leakage current [45,46]. Therefore, it was confirmed that when the thickness of thin films initially deposited using RP was 5 nm or more, the ion bombardment effect on the TO was reduced, resulting in decreased interface defect density and increased device endurance, as shown in Figure 3. Figure 8b presents the memory retention characteristics, which were obtained to investigate the memory performance of the fabricated CTM device. A voltage of ±8 V was applied for 1 s, and the memory window was observed over time from 10^0^ to 10^3^ s to predict the memory characteristics of the device after 10 years [47]. During the 10-year retention period, the memory window of the thin film deposited using the sequential RP and DP processes showed an intermediate value between those of the DP-only and RP-only films. However, the memory window decay rate showed no significant variation with the thickness ratio of the thin film layers. This is believed to be because the degradation of retention characteristics is more influenced by the quality of the TO than by the interface defect density. Therefore, a wide memory window value of 3.8 V was maintained even after 10 years, irrespective of the charge loss, particularly when the thickness ratio of the sequential RP and DP-deposited thin film was RP(7):DP(3) [48,49].

The results comparing the memory characteristics of the CTM device fabricated in this study with previously published research findings are presented in Table 1. The CTM device produced in this study exhibited superior memory window characteristics, even at lower annealing temperatures and operating voltages, compared to existing CTM device characteristics, indicating significant improvements.

## 4. Conclusions

In this study, a new ALD process that involved the sequential deposition of RP and DP was employed to deposit an HfO_2_ CTL. We meticulously investigated the effect of the interface characteristics of Si/SiO_2_/HfO_2_ on the operational characteristics of CTM devices. As the thickness of the initially RP-deposited thin film increased, the memory window increased, and the interface trap density (D_it_) decreased. Specifically, when the RP-deposited thin film thickness was 7 nm, the memory window reached a maximum value of 10.1 V at an operating voltage of ±10 V, and D_it_ reached a minimum value of 1.0 × 10^12^ eV^−1^cm^−2^. XPS analysis results confirmed that the thin film deposited using the sequential RP and DP processes exhibited a lower non-lattice ratio, indicating defects at the Si/SiO_2_/HfO_2_ interface compared to the thin film deposited using only DP. This suggests that using RP reduces interface defects. Additionally, when the RP-deposited thin film thickness was higher than or equal to 5 nm, the device endurance significantly increased, and the V_FB_ shift results also showed improved charge trapping efficiency. This suggests that during DP thin-film deposition, ion bombardment affects the substrate up to approximately 5 nm depth. When the thickness of the RP-deposited thin film was 5 nm or more, the ion bombardment effect of DP on the TO decreased, improving endurance and reliability.

Consequently, the HfO_2_ thin film deposited using the sequential RP and DP processes exhibited overall improved electrical characteristics. These results confirm that the sequential plasma deposition process can solve substrate damage and unstable interface layer issues, improving thin film characteristics and enhancing device performance. Moreover, by applying the DP process alternately with the RP process, which has a long process time, the deposition rate increased and the overall process time shortened, confirming the effect of increased throughput. In the future, it is anticipated that this process technology can be applied to various structures to develop memory devices with superior performance and reliability.

## Figures and Tables

**Table 1:** Comparison among the memory characteristics of high-k-based CTM devices.

TO/CTL/BO	Thickness (nm)	Annealing temp. (˚C)	Operating Voltage (V)	Memory Window (V)	Charge Loss (%)	References
SiO_X_/RP:DP-HfO_2_/Al_2_O_3_	2/7:3/9	400	±10	10.1	37	This work
SiO_X_/RP-HfO_2_/Al_2_O_3_	2/9/9	400	±12	12.66	34.32	[16]
SiO_2_/HfO_2_/Al_2_O_3_	3/10/10	1000	±15	7.4	31	[50]
SiO_2_/HfAlO/Al_2_O_3_	3/9/8	800	±16	11.5	14.9	[51]
Al_2_O_3_/HfAlO/Al_2_O_3_	2/9/12	600	±12	6.29	79	[52]
SiO_2_/ZrO_2_/Al_2_O_3_	5/10/15	700	±11	7.1	16	[53]

## Data Availability

The data presented in this study are contained within the article.
